# Safety profile of Coartem^®^: the evidence base

**DOI:** 10.1186/1475-2875-8-S1-S6

**Published:** 2009-10-12

**Authors:** Catherine Falade, Christine Manyando

**Affiliations:** 1 Department of Pharmacology and Therapeutics, College of Medicine, University of Ibadan, Ibadan, Nigeria; 2 Tropical Diseases Research Centre, P.O. Box 71769, Ndola, Zambia

## Abstract

This article reviews the comprehensive data on the safety and tolerability from over 6,300 patients who have taken artemether/lumefantrine (Coartem^®^) as part of Novartis-sponsored or independently-sponsored clinical trials. The majority of the reported adverse events seen in these studies are mild or moderate in severity and tend to affect the gastrointestinal or nervous systems. These adverse events, which are common in both adults and children, are also typical of symptoms of malaria or concomitant infections present in these patients. The wealth of safety data on artemether/lumefantrine has not identified any neurological, cardiac or haematological safety concerns. In addition, repeated administration is not associated with an increased risk of adverse drug reactions including neurological adverse events. This finding is especially relevant for children from regions with high malaria transmission rates who often receive many courses of anti-malarial medications during their lifetime. Data are also available to show that there were no clinically relevant differences in pregnancy outcomes in women exposed to artemether/lumefantrine compared with sulphadoxine-pyrimethamine during pregnancy. The six-dose regimen of artemether/lumefantrine is therefore well tolerated in a wide range of patient populations. In addition, post-marketing experience, based on the delivery of 250 million treatments as of July 2009, has not identified any new safety concerns for artemether/lumefantrine apart from hypersensitivity and allergies, known class effects of artemisinin derivatives.

## Background

Malaria is one of the most significant causes of morbidity and mortality worldwide, causing approximately 881,000 deaths every year [1]. The current WHO guidelines for the treatment of malaria recommend the use of artemisinin-based combination therapy (ACT) owing to the rising threat of *Plasmodium falciparum *resistance to monotherapy [2]. Artemether/lumefantrine (AL) was the first fixed-dose combination of ACT to be approved by the European regulatory authorities according to the requirements of the International Committee on Harmonization (ICH). AL is also pre-qualified by WHO for efficacy, safety and quality and is marketed as Coartem^® ^for use as a six-dose regimen in infants, children and adults with acute, uncomplicated infection due to *P. falciparum *or mixed infections including *P. falciparum *[3,4].

The efficacy of AL has been confirmed in many different patient populations around the world and is discussed elsewhere in this supplement [5]. This article reviews the comprehensive safety data on AL that has been reported in the scientific literature from Novartis-sponsored and independent clinical trials.

## Novartis-sponsored studies

Six Novartis-sponsored clinical studies [6-11] have been conducted to assess efficacy and safety of the six-dose regimen of AL, with safety data from these summarized in Table 1. These studies were conducted in different regions of the world and so included patients who lived in areas with varying levels of drug resistant *P. falciparum *and malaria endemicity. Some of the studies included other anti-malarial drugs as active comparators [7,8], one allowed the inclusion of patients with mixed infections that included *P. falciparum *[10], and one utilized a dispersible paediatric formulation [11].

**Table 1:** Key clinical studies evaluating the safety of the six-dose regimen of AL.

**Study number**	**Design**	**Comparator**	**Patients**	**Number given AL compared with total**	**Participating countries**	**Key safety findings**
van Vugt et al 1999 [6]	Randomized, double-blind	Six-dose regimen	Adults & children (>2 years)	120 out of 359^†^	Thailand	Possible treatment-related AEs were reported by 16.7% of patients. These AEs could also have been related to malaria and were mild or moderate in severity. No neurological or cardiac safety issues were identified.
van Vugt et al 2000 [7]	Randomized, open-label	MAS*	Adults & children (≥ 2 years)	150 out of 200	Thailand	Most AEs considered drug-related were mild or moderate in severity. Possibly related AEs occurred at a frequency of 22% in the AL group compared with 46% in the MAS group. In the AL group 6% had nervous system AEs compared with 34% in the MAS group (relative risk: 0.18; p < 0.0001). Gastrointestinal AEs were also less frequent in the AL compared with MAS groups (12.7% vs 26%; relative risk: 0.49; p < 0.043). No ECG abnormalities were identified.
Lefèvre et al 2001 [8]	Randomized, open-label	MAS*	Adults & adolescents (≥ 12 years)	164 out of 219	Thailand	Nearly 90% in both treatment groups reported treatment emergent signs/symptoms and these were of mild or moderate severity. 18.3% of patients taking AL and 21.8% of patients taking MAS reported gastrointestinal symptoms while 27.4% and 16.4% of patients, respectively reported headache, dizziness and sleep disorder. Skin reactions were reported by 4.9% of patients taking AL and 3.6% of patients taking MAS. There were no cardiac complications and no significant renal, hepatic or haemopoietic dysfunction.
Falade et al 2005 [9]	Open-label	Nil	Infants & children (5 to 25 kg)	310	Kenya, Tanzania, Nigeria	The most commonly reported AEs were cough, anaemia, anorexia, vomiting and diarrhoea. Some differences in AEs were seen in the different body weight groups but as these were generally mild, differences were not considered clinically relevant. Only one patient had a SAE (urticaria) that was considered to be related to study medication and this event resolved when treatment was withdrawn. No cardiac safety issues were identified and there were no significant abnormal laboratory values associated with AL treatment.
Hatz et al 2008 [10]	Open-label	Nil	Adult, non-immune travellers	165	EU, Colombia	Treatment was well tolerated and most AEs were mild or moderate in severity. The most frequently reported AEs were headache, insomnia, diarrhoea, nausea and vomiting and these AEs (along with anorexia, vertigo and chills) were most probably related to signs and symptoms of malaria. No allergic reactions were reported in any of the patients. There were few SAEs and none of these were considered related to AL. No significant effects were observed during ECGs and there were no significant effects seen with regard to laboratory parameters.
Abdulla et al 2008 [11]	Randomized, investigator-blind	Dispersible formulation of AL	Infants & children (5 to <35 kg)	452 out of 899	Kenya, Tanzania, Mali, Benin, Mozambique	There was no difference in the pattern of AEs seen in patients who received treatment with crushed AL tablets compared with those who received the dispersible formulation. No new or unexpected AEs were identified and the most commonly reported AEs were also related to malaria. The most common drug-related AE was vomiting; this was more frequently reported in the lowest weight category. The number of SAEs was low (1-2% in both groups) and most of these were infections. There were no signs of ototoxicity. There were no clinically relevant findings or differences between study groups for ECG assessments, vital signs, or laboratory parameters.

*Study was not designed to compare artemether/lumefantrine with mefloquine/artesunate

^†^Artemether/lumefantrine over 60 hours

AE: adverse event;AL: artemether/lumefantrine; ECG: electrocardiogram; MAS: mefloquine/artesunate; SAE: serious adverse event; vs.: versus

In all of these studies AL was well tolerated and most reported adverse events were of mild or moderate severity. Reported adverse events occurred mainly in the nervous system or gastrointestinal system and were considered typical of the symptomatology of malaria or concomitant infections. No cardiac, neurological or audiological safety issues were identified in the studies that assessed these safety parameters [6-11].

## Comparative results from independent studies

Many independent trials on AL have also been published in the scientific literature. Results from the studies conducted in Africa [12-32] are summarized in Table 2 and the results from studies conducted in Asia [33-39] in Table 3. The safety findings from these trials confirm the results of the Novartis-sponsored studies, namely that the majority of adverse events were mild or moderate in severity, tended to affect the gastrointestinal or nervous system, and were consistent with the symptoms of malaria. Serious adverse events were infrequent and were unrelated or unlikely to be related to study medication.

**Table 2:** Published studies with the six-dose AL regimen in Africa

**Authors**	**Country**	**Design and patient population**	**Comparators (no. of patients)**	**Results**
Bukirwa et al 2006 [12]	Uganda	Randomized, single-blind, single centre in children (1-10 years)	AL (n = 204) ASAQ (n = 204)	Overall, 261 (65%) of participants experienced AEs of moderate or severe intensity and there was no difference between the two treatment groups. SAEs were uncommon with both regimens, with one occurring in each group and judged to be unrelated to study medication (ASAQ) or unlikely to be related to study medication (AL). No abnormalities in hearing or fine finger dexterity were detected.
Dorsey et al 2007 [13]	Uganda	Single-blind, randomized, single centre in children (1-10 years)	AL (n = 103 and202 treatments) AQSP (n = 111 and 253 treatments) ASAQ (n = 113 and 232 treatments)	All study regimens appeared to be safe and generally well tolerated. In the first 14 days, anorexia and weakness occurred more commonly in children treated with AQSP than those receiving ASAQ or AL. A total of 45 SAEs were reported in 38 patients, but none of these were considered probably or definitely related to study medication.
Gürkov et al 2008 [14]	Ethiopia	Single centre in adults and children >5 years of age	AL (n = 30) Quinine (n = 35) Atovaquone/proguanil (n = 32)	The first randomized clinical trial to directly compare ototoxicity of AL with other anti-malarial drugs. Pure tone audiometry and distortion product otoacoustic emission levels revealed transient significant cochlear hearing loss in patients treated with quinine but not in those treated with AL or atovaquone/proguanil. Transitory evoked otoacoustic emission could be elicited in all examinations, except for three patients in the quinine group on day 7, who suffered a transient hearing loss greater than 30 dB. There was no evidence of drug-induced brain stem lesions by brain stem evoked response audiometry. There was no detrimental effect of AL on peripheral hearing or brainstem auditory pathways; however, transient hearing loss is common after quinine therapy due to temporary outer hair cell dysfunction.
Kamya et al 2007 [15]	Uganda	Single-blind, randomized, single centre in children (6 months to 10 years)	AL (n = 210) DP (n = 211)	Both drugs were well tolerated and reported AEs were of mild or moderate severity and consistent with symptoms of malaria. There were 6 SAEs reported in this study and all were judged unrelated to study medications.
Mårtensson et al 2005 [16]	Zanzibar	Multicenter, randomized, open-label in children (6-59 months)	AL (n = 200) ASAQ (n = 208)	Both drugs were well tolerated and most AEs were of mild severity. Similar proportions of moderate or severe AEs were reported by the two groups (10% for AL vs. 12% for ASAQ). All severe AEs were associated with malaria and were not attributed to the study drugs. No significant differences were seen in the mean counts of white blood cells and neutrophils during follow-up. Significant and similar increases in mean haemoglobin levels from baseline to day 42 were seen in both groups.
Mohamed et al 2006 [17]	Sudan	Two-centre, open-label in children and adults	AL (n = 72) ASSP (n = 71)	No AEs were recorded for patients given ASSP and 11 were recorded for patients given AL (5 cases of gastric disturbance, 5 of excessive sleepiness and 1 of dizziness). All were mild and self-limited and no patients withdrew from the study because of an AE.
Yeka et al 2008 [18]	Uganda	Randomized, single centre, single-blinded study in children aged 6 months to 10 years	AL (n = 199) DP (n = 215)	Most AEs were of mild or moderate severity and consistent with symptoms of malaria. The most commonly reported AEs in both groups were cough, coryza, abdominal pain, anorexia, weakness, diarrhoea and pruritus and there were no significant differences between groups. A total of 7 SAEs were reported (2.3% after DP and 1% after AL), but all were judged unrelated to study medication.
Mulenga et al 2006 [19]	Zambia	Randomized, open-label, multicentre in adults	AL (n = 485) SP (n = 486)	AL and SP were generally well tolerated. SAEs were observed in 2 patients treated with SP and one patient treated with AL. Eleven patients (5 on AL and 6 on SP) had various symptoms possibly related to the study drugs but none were serious enough to interrupt the treatment.
Adjei GO et al 2008 [20]	Ghana	Randomized, open label in children (6 months to 14 years)	AL (n = 111) ASAQ (n = 116)	The majority of AEs were mild in intensity, overlapped with known malaria symptomology, and were mostly classified as unrelated to study medication. Drug-related AEs were rare. Possibly related AEs were pruritus (2 patients on ASAQ and 1 patient on AL) and fatigue/excessive sleepiness (5 patients on ASAQ and 4 patients on AL). Neurological examinations were conducted on 168 children (n = 92 for ASAQ; n = 76 for AL). Nystagmus was observed in two patients (1 per group), but both had a history of admission to a neonatal intensive care unit and either excessive emotional liability or cognitive impairment. One child in the AL group had a positive Romberg's test (indicative of sensory ataxia) on day 3, but also had a history of cognitive impairment. No other neurological abnormalities were observed during the 28-day follow-up, at monthly visits or in patients who took multiple courses of treatment. Audiometry was assessed in 72 patients (n = 37 for ASAQ; n = 35 for AL). Hearing thresholds were significantly elevated in treated patients compared with age- and sex-matched controls up to and including day 28, but there were no differences after 9 to 12 months. (Note: full audiometry results are presented elsewhere).
Falade et al 2008 [21]	Nigeria	Randomized, open label, single centre in children (6 months to 10 years)	AL (n = 66) ASAQ (n = 66)	Both drugs were well tolerated according to clinical and laboratory parameters. The most common AEs were vomiting, anaemia, cough and abdominal pains and are frequent clinical findings among patients with malaria. No child was withdrawn because of an AE.
Faye et al 2007 [22]	Senegal	Randomized, open-label, multicentre in children and adults	ASAQ (n = 360) AQSP (n = 161) MAS (n = 145) Six-dose AL (n = 149) Four-dose AL (n = 140)	The side-effects of treatments were minor and consisted mainly of mild gastralgia, dizziness, pruritus, asthenia, and vomiting. These disappeared at the end of treatment and did not require any specific treatment. No SAEs were observed and there were no severe alterations in renal or hepatic function for any of the drug combinations.
Koram et al 2005 [23]	Ghana	Two-centre, randomized, open-label in children (6-59 months)	AL (n = 51) CQ (n = 36) SP (n = 27) ASAQ (n = 54)	Some patients were withdrawn from the study due to AEs: 2 patients on day 1 due to lethargy/inability to eat, (1 each for the CQ and SP groups), 3 patients due to excessive vomiting/lethargy (all after ASAQ) and 1 patient due to excessive vomiting and diarrhoea (this patient in the AL group had a concomitant hookworm infection).
Owusi-Agyei et al 2008 [24]	Ghana	Randomized, open label in children aged 6 months to 10 years	AL (n = 223) ASAQ (n = 228) ASCD (n = 178)	The incidence of AEs was comparable between the groups although a history of body pain was more frequently reported in the ASAQ group compared with the AL or ASCD groups (14.0% vs. 5.7% vs. 5.1%, respectively; p = 0.01).
Sagara et al 2006 [25]	Mali	Randomized, single centre, open-label study in children (≥ 6 months) and adults	AL (n = 303) AS plus sulphamethoxypyrazine plus pyrimethamine (n = 303)	AL and AS plus sulphamethoxypyrazine plus pyrimethamine were both well tolerated. The total number of reported AEs and the number of patients reporting any symptom/signs in the first week after treatment was similar in the groups. Diarrhoea occurred more frequently after AL compared with AS plus sulphamethoxypyrazine-pyrimethamine (3.6% vs. 1.0%, respectively; p = 0.03). No SAEs occurred.
Sowunmi et al 2007 26]	Nigeria	Randomized, open-label, single centre, in children (≤ 10 years)	AL (n = 90) AQ plus sulphalene plus pyrimethamine (n = 91)	AEs within the first week of treatment were reported by 21 children (9 after AL and 12 after AQ plus sulphalene plus pyrimethamine). There was no significant difference in the proportions of patients reporting AEs in both groups. Pruritus and weakness were significantly more frequent in the AQ plus sulphalene plus pyrimethamine group, and vomiting was significantly more frequent in the AL group. No child was withdrawn because of drug intolerance.
Sutherland et al 2005 [27]	Gambia	Randomized, single centre, single-blind, in children (1-10 years)	AL (n = 406) CQSP (n = 91)	Minor complaints noted at the day 7 laboratory visit included headache (12% and 11% in the CQSP and AL groups, respectively), anorexia (12% and 16%), diarrhoea (7% and 4%), abdominal pain (5% and 5%), and pruritus (1% and 1%). No SAEs occurred in either group, and there were no deaths among children in the study.
Zongo et al 2007 [28]	Burkina Faso	Multicentre, randomized, open-label in children (6 months to 10 years)	AL (n = 261) AQSP (n = 260)	Both drugs were well tolerated. AEs did not differ between groups apart from pruritus, which was more common with AQSP than AL (16% vs. 3%, p < 0.0001). Only 2 SAEs (both haemoglobin levels below 50 g/L) were reported; 1 SAE was due to early treatment failure after AQSP and 1 SAE was due to late clinical failure after AL.
Zongo et al 2007 [29]	Burkina Faso	Multicentre, randomized, open-label in children (≥ 6 months)	AL (n = 188) AQSP (n = 184) DP (n = 187)	All drugs were well tolerated. However, abdominal pain was reported more often in the AQSP and AL groups than in the DP group (24% and 20% vs. 9%, respectively), headache was more common in the AL group than in the AQSP and DP groups (21% vs. 14% and 10% respectively) and pruritus was more common in the AQSP group than in the AL and DP groups (18% vs. 6% and 3%, respectively). No SAEs were reported in this study.
Fanello et al 2007 [30]	Rwanda	Randomized, open-label, 2-centre in children (12-59 months)	AL (n = 251) AQSP (n = 249)	AEs concomitant with the administration of the study drug were reported by 251 patients (52.21% after AQSP and 48.21% after AL). AEs that were possibly or probably related to the study drug were reported by 22.73% of patients after AQSP and 14.47% after AL (p = 0.06). The most frequently reported AEs after AQSP were fatigue, anorexia, vomiting and abdominal pain and the AEs most frequently reported after AL were cough and diarrhoea.
Ndayiragije et al 2004 [31]	Burundi	Multicentre, randomized, open-label in children (<5 years)	AL (n = 142) ASAQ (n = 153)	AEs were similar in the two groups, although vomiting on days 1 and 2 was more frequent in the ASAQ group (13.1% and 5.3%, respectively) than in the LA group (5% and 0.7%, respectively).
Van den Broek et al 2006 [32]	Republic of Congo	Randomized, single centre, open-label study in children (6-59 months)	AL (n = 106) ASAQ (n = 101) ASSP (n = 91)	The most frequent AEs reported during the 3 days of treatment were vomiting, diarrhoea, abdominal pain and anorexia. The frequency of these AEs was low (around 10% of children) and did not differ among the groups. There were two cases of urticaria (1 after ASAQ and 1 after ASSP), but these developed after completion of the treatment. No SAEs were reported.

AL: Artemether/lumefantrine; CQ: Chloroquine; SP: Sulphadoxine/pyrimethamine; CQSP: Chloroquine/sulphadoxine/pyrimethamine; AS: Artesunate; AQ: Amodiaquine; ASAQ: Amodiaquine/artesunate; AQSP: Amodiaquine/sulphadoxine/pyrimethamine; DP: dihydroartemisinin/piperaquine; MAS: mefloquine/artesunate

**Table 3:** Published studies with the six-dose AL regimen in Asia

**Authors**	**Country**	**Design and patient population**	**Comparators (no. of patients)**	**Results**
*South-East Asia*				
Krudsood et al 2003 [33]	Thailand	Randomized, open-label, single centre in adults	AL (n = 41) DNP (n = 89)	No deterioration in clinical or biochemical responses occurred after treatment with AL or DNP. Minor symptoms (nausea, headache, and dizziness) were seen in both groups. These could not be differentiated from malaria signs and symptoms as they resolved simultaneously with fever 1-4 days after treatment. There were no SAEs or deaths, and no neurological or neuropsychiatric manifestations were seen during treatment or during the 28-day follow-up period.
Stohrer et al 2004 [34]	Laos	Randomized, open-label, hospital and community-based study in adults and children (≥ 10 kg)	AL (n = 53) MAS (n = 55)	Most of the recorded treatment emergent symptoms/signs (TESS) on day 0 and day 3 were mild or moderate in severity, and were symptoms typical of malaria. There were no significant differences between treatment groups in the incidence of gastrointestinal disorders like abdominal pain, nausea, vomiting, diarrhoea or anorexia (12.8% for AL vs. 12.0% for MAS), or nervous system disorders like headache, dizziness, weakness, or sleep disorder (29.8% for AL vs. 41.5% for MAS). Apart from severe diarrhoea (1 patient in the AL group) and sleep disorder and dizziness (1 patient in the MAS group), no other potentially drug-related AEs were reported.
Mayxay et al 2004 [35]	Laos	Randomized, open-label, single centre, in adolescents and adults (12-19 years)	AL (n = 110) MAS (n = 110) CQSP (n = 110)	The proportion of patients with symptoms and signs before treatment, which may subsequently be confused with drug-related AEs, did not differ significantly between the 3 groups. The proportion of patients with ≥ 1 potential side-effect was higher in the MAS group (52%) than in the CQSP group (44%) or the AL group (27%). Three patients in the MAS group had serious neuropsychiatric effects following treatment, 1 patient developed fever and a Glasgow coma score of 8/15 due to the presence of gametocytes on day 15 after CQSP and 1 patient had hallucinations and uncharacteristic anxiety on day 20.
Ratcliff et al 2007 [36]	Indonesia	Randomized, open-label, 2-centre in children (body weight ≥ 10 kg) and adults	AL (n = 387) DP (n = 387)	AEs were assessed in patients without the symptom at enrolment and were similar between groups apart from a two-fold increased risk of diarrhoea on days 1 and 2 in more patients receiving DP compared with AL (95% CI 1.3-3.3; p = 0.003). By day 7, the risk of diarrhoea was 5% in both treatment groups. Although 35% of patients developed a headache on days 1 and 2 after DP compared with 23% of patients given AL, the difference was not significant as headache was a common symptom at presentation.
Hutagalung 2005 [37]	Thailand	Randomized, open-label, 2-centre, in children (>10 kg) and adults	AL (n = 245) MAS (n = 245)	AL and MAS were well tolerated. Vomiting of one or more doses of drug occurred in 2.1% in the AL group and 0.8% of the MAS group (relative risk, 2.5; 95% CI, 0.5-12.7; p = 0.45). The rates of early vomiting (within one hour) of dosing were very low (around 2%) and did not differ between groups. The most commonly reported and possibly drug-related AEs were effects on the gastrointestinal (abdominal pain, anorexia, nausea, diarrhoea and vomiting more than 1 hour after dosing) and central nervous system (headache, dizziness). There were fewer AEs in the AL group compared with the MAS group, although this was not statistically significant. No SAEs were reported.
*South Asia*				
van den Broek et al 2005 [38]	Bangladesh	Randomized, open-label, single centre, in adults and children (≥ 1 year)	AL (n = 121) MAS (n = 121) CQSP (n = 122)	All treatments were well tolerated. Mild AEs reported during the 3 days of treatment were headache, vomiting, nausea and dizziness. The frequency of these AEs was generally higher after MAS compared with AL (p < 0.05). After CQSP treatment, complaints were of intermediate frequency, but vomiting occurred more in this group. Other AEs were anorexia, skin itching and deafness with CQSP, sleeplessness, anorexia, skin itching/rash, epigastric pain and excessive sweating with MAS and blurred vision and anorexia with AL. No severe AEs were observed.
Thapa et al 2007 [39]	Nepal	Randomized, open-label, single centre, in adults and children (>5 years)	AL (n = 66) SP (n = 33)	The most commonly reported symptoms at presentation apart from fever were headache (97% in the AL group and 88% in the SP groups), nausea (42% and 64%, respectively), and vomiting (39% and 46%, respectively). Other gastrointestinal, neurological, musculoskeletal, respiratory, and dermatologic complaints were much less frequent. During treatment, <12.5% of patients reported one or more symptoms, with the majority of mild intensity, and no significant differences seen between groups. There were no group-specific differences in changes in pulse or blood pressure during initial therapy. ECGs were conducted in 10 patients in the AL group and 8 patients in the SP group and no changes in QTc were observed during treatment with either drug.

AL: artemether/lumefantrine; ECG: electrocardiogram; SP: sulphadoxine/pyrimethamine; CI: confidence interval; CQSP: Chloroquine/sulphadoxine/pyrimethamine; DP: Dihydroartemisinin/piperaquine; DNP: dihydroartemisinin/napthoquine/trimethoprim; MAS: mefloquine/artesunate; vs.: versus

Data have also been pooled to provide a more complete assessment of the safety and tolerability of AL in a large number (>1,900) of adult and paediatric patients. The results of two of these meta-analyses, Mueller *et al *and Makanga *et al *are shown in Table 4[40,41]. These pooled results confirm earlier data and show that AL is generally well tolerated with the majority of reported adverse events again affecting either the gastrointestinal or nervous systems and of mild or moderate severity [40,41]. Few adverse events were considered by the investigators to be drug-related - most were regarded as being related to the symptomatology of malaria. Serious adverse events occurred infrequently [40,41]. There were, however, some differences in the adverse event profile in children when different body weights were compared, but this is unlikely to be of clinical relevance. Instead, it is more likely to be related to the subjective nature of these adverse events and the differences in the ability of young infants and children to verbalize these complaints [41].

**Table 4:** Pooled analyses of data on the six-dose AL regimen

**Authors**	**Number of studies, regions, types of patient and drugs (number of patients)**	**Results**
Mueller et al 2006 [40]	11 randomized clinical trials conducted in Thailand, India, Europe, China, Bangladesh in adolescents and adults aged >12 years	Almost every patient reported at least one AE during treatment. The rate and type of AEs were generally comparable between the 6- and four-dose groups. Most AEs were reported during the first 3 days of treatment and were of mild or moderate severity. Severe AEs were infrequent (5.9% for the six-dose group, 3.8% for the four-dose group). The most common AEs reported for AL included headache, asthenia, dizziness, myalgia, arthralgia, nausea, anorexia and fatigue; all of these could have been disease-related. Only a small proportion of patients reported AEs that were suspected to be drug-related and these occurred less frequently with the six-dose AL group than in the four-dose group. Drug-related AEs were more frequent for most comparator treatments than six-dose AL regimen, and for MAS, these were higher than the rates seen in both the six-dose and four-dose groups.
	Six-dose AL (n = 598) Four-dose AL (n = 770)	
	Comparators included: Mefloquine (n = 126) Quinine/SP (n = 114) Chloroquine (n = 90) MAS (n = 335) Halofantrine (n = 52)	
		SAEs were reported by 0.6% of patients in the six-dose group and 0.8% of patients in the four-dose group. No SAEs in patients in the six-dose group (dyspnoea, febrile coma, pulmonary oedema, typhoid fever) were considered to be drug-related. SAEs in the four-dose group included (one each of) abnormal laboratory values, anaemia, falciparum malaria, malaria relapse, severe malaria, and two cases of hepatitis; only the reports of anaemia and malaria relapse were suspected to be drug-related. For the comparators, one SAE was reported in each of the MAS and chloroquine groups.
		There was no difference in neurotoxicity between any of the groups, apart from paresthesia, which was only present in the four-dose AL group and the MAS group. Headache and dizziness were the most frequently reported neurological AEs. Decreased hearing (hypoacusis) was reported by 1.6% of patients in the four-dose group; there were no reports in the six-dose group. All cases of hypoacusis were mild, apart from one case of moderate severity and only two were considered to be drug-related. For the comparators, only patients in the MAS group reported hypoacusis (6.3%).
Makanga et al 2006 [41]	8 clinical trials in Gambia, Tanzania, Kenya, Nigeria and Thailand in children (5-25 kg)	The majority of patients reported at least one AE after dosing; these were generally mild or moderate in severity. Severe AEs were infrequent (5.2% for the six-dose and 7.0% for the four-dose AL groups). The most commonly reported AEs included cough, anaemia, anorexia, vomiting, hepato-/splenomegaly, headache, and diarrhoea, with most of these possibly related to malaria. The proportion of patients with AEs was similar between the 5 to <10, 10 to <15, and 15-25 kg body weight groups for both dosing groups. There were some differences between body weight groups for certain AEs such as headache and dizziness. These subjective AEs appeared less commonly in very small infants, but this may have been related to the patients' limited ability to verbalize symptoms and as such is not considered clinically relevant. Drug-related AEs were only reported for a small number of patients and occurred more frequently in the six-dose group.
	Six-dose AL (n = 343) Four-dose AL (n = 201)	
		Only three patients (0.9%) in the six-dose group reported SAEs (convulsion, urticaria, viral hepatitis) compared with one patient (0.5%) in the four-dose group (pneumonia). Of these SAEs, only the case of severe urticaria, which required hospitalisation, was considered drug-related. In addition to these SAEs, one patient died, but this was from causes unrelated to study treatment. Fewer patients in the six-dose than in the four-dose group reported AEs that were related to the central nervous system; headache (9.9% vs. 40.3%, respectively) and dizziness (3.8% vs. 16.4%, respectively) were the most commonly reported events. Other neurological AEs reported in the six-dose and four-dose groups included clonus (3.8% vs. 1.0%, respectively), hyperreflexia (1.7% vs. 0.5%, respectively), and convulsions (0.3% vs. 1.0%). Patients in the six-dose group failed to report the following AEs which were seen in the four-dose group: nystagmus, ataxia, and coordination abnormal (all 1.0-1.5%) and decreased hearing (1.5%). The cases of decreased hearing were not considered to be drug-related.
		For cardiac safety assessments, the incidence of QTc changes within specific ranges was comparable between groups, and it is unlikely that there is an increased cardiac risk in the paediatric patients included in this analysis. Clinical laboratory parameters showed no major differences between the groups; findings were consistent with acute malaria.

AE: adverse event;AL: artemether-lumefantrine; MAS: mefloquine plus artesunate; SAE: serious adverse event; SP: sulphadoxine plus pyrimethamine vs.: versus

## Special safety considerations

### Neurological and audiological safety

There have been case reports of neurological problems (including ataxia, nystagmus, tremor and slurred speech) occurring after administration of herbal artemisinin [42] or artesunate monotherapy [43,44]. However, it is questionable whether these neurological effects are related to artemisinin treatment [45-47]. Indeed, this is not supported by audiology case-control [48-50] or other audiology data [14,51], although additional analyses are often called for to investigate this fully. There were, however, no neurological or audiological safety concerns identified for AL in the Novartis-sponsored studies [6-10,52] as shown in Table 1. In addition, audiological data are currently being analysed from a Novartis-sponsored study in 265 patients treated with AL, malarone or mefloquine-artesunate. [Personal communication with Novartis, study A2417). A routine review of the data collected from the first 85 patients, however, did not raise any safety concerns. Neurological or audiological safety concerns have not been identified in the African studies by Bukirwa *et al *[12], Gürkov *et al *[14], and Adjei *et al *[20], as shown in Table 2, Asian studies by Krudsood *et al *[33], and Mayxay *et al *[35], as shown in Table 3, and in the pooled analyses reported by Mueller *et al *[40] or Makanga *et al *[41], which are summarized in Table 4.

### Cardiac safety

Lumefantrine is chemically related to halofantrine, an anti-malarial known to be associated with significant prolongation of QTc interval. Indeed, QTc prolongation is a known class effect of many anti-malarial drugs. As such, cardiac safety has been thoroughly investigated during the preclinical and clinical development of AL.

**Figure 1:**
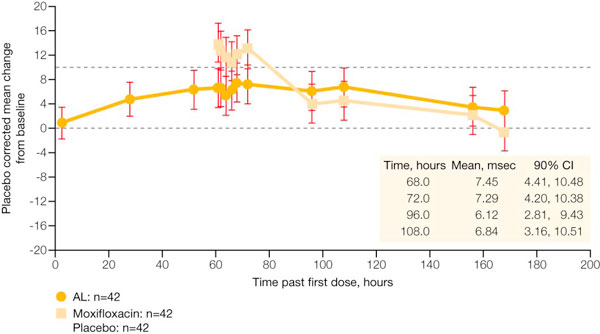
**Placebo-corrected mean change in QTc (Fridericia's formula) from time-averaged baseline in healthy volunteers**. [Data on file (study A2101), Novartis]. AL = artemether/lumefantrine; CI = confidence interval.

**Figure 2:**
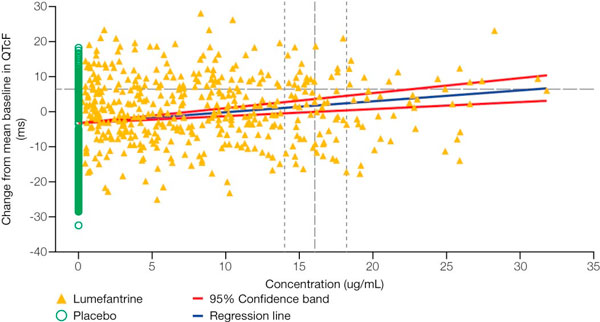
**Relationship between QTc (Fridericia's formula) and maximum plasma concentration (C_max_) in healthy volunteers**. [Data on file (study A2101), Novartis]. Note: the threshold of relevance for QTc change is 10 msec vs. placebo.

The effects of lumefantrine and its major metabolite desbutyl-lumefantrine on wild-type hERG K^+ ^channels have been investigated in stably transfected human embryonic kidney cells (HEK293) using a whole cell patch-clamp technique [52]. This *in vitro *hERG channel assay showed that lumefantrine and desbutyl-lumefantrine have higher IC_50 _values (approximately 200-fold) than halofantrine (see Table 5). In addition, the calculated cardiac safety indices, which are over 30 for lumefantrine, suggest that lumefantrine and its major metabolite pose an unlikely risk of cardiotoxocity compared with halofantrine [52]. A phase I, parallel group study with AL, moxifloxacin and placebo arms (n = 42 per group) was conducted in healthy volunteers to assess cardiac safety [52]. As shown in Figure 1, this study demonstrated that the six-dose regimen of AL was associated with a mean maximum increase in QTc (Fridericia's formula) of 7.45 msec compared with placebo (90% CI: 4.4 to 10.5 msec) in healthy volunteers. By comparison, the effect on QTc (Fridericia's formula) was much greater after dosing with moxifloxacin, the positive control. A formal analysis of the relationship between the plasma concentration of lumefantrine and the change from mean baseline in QTc (Fridericia's formula) was conducted (see Figure 2). This shows that the 95% confidence limit for the mean lumefantrine plasma concentration does not cross the upper confidence band for the threshold of relevance for change in QTc (Fridericia's formula).

**Table 5:** *In vitro *IC_50 _for hERG tail current in HEK 293 cells and cardiac safety index of antimalaria drugs [52]

**Agent**	**IC_50 _- IK_r _(μM)**	IC_50_/therapeutic free plasma concentration*
Halofantrine	0.04	0.07
Chloroquine	2.5	6.3
Mefloquine	2.6	50
Desbutyl-lumefantrine	5.5	~2,900
Lumefantrine	8.1	48

* Cardiac safety index. Values greater than 30 suggest cardiotoxicity is unlikely

hERG: human ether-a-go-go related gene; IC_50_: inhibitory concentration 50 (the concentration that gives 50% inhibition)

One possible explanation for the alterations in QTc seen with AL may be due to malaria itself and recovery from this disease, as well as to stress and anaemia. These conditions can affect cardiac electrophysiology and may lengthen the QT interval [53]. As QT correction formulae are based on a normal heart rate of 60 beats/minute, and patients with malaria tend to have elevated heart rates that decrease with successful treatment and defervescence, overcorrection of the QT interval can also occur. This is even more pronounced in small children, and heart rates higher than 60 beats/minute are routinely seen in healthy children. Despite the changes in QTc associated with malaria, no adverse events attributable to QTc prolongation (e.g. syncope or sudden death) have been reported in clinical trials with AL [6] as shown in Table 1[6-11], Table 3[39], and Table 4[40,41].

### Haematological safety

Preclinical data suggested that repeated exposure to AL may affect blood cell counts and as such, particular attention was paid to haemoglobin levels and haematological adverse events reported during clinical trials. A published pooled analysis of 15 trials conducted in China, India, Thailand, The Gambia, Tanzania, France, The Netherlands and the UK confirmed these results [54]. Indeed, in this analysis, anaemia and thrombocytopaenia were frequently present at baseline, but returned to normal or improved considerably with the resolution of disease [54]. No haematological safety concerns were identified during the safety assessment presented in this review.

## Safety of repeated administration

Most safety data on antimalarials are collected as part of clinical trials and these tend to evaluate the treatment of a single episode of malaria. In practice, however, children in areas of high malaria endemicity are likely to suffer from repeated episodes of malaria and this raises concerns over potential toxicity due to repeated short-term exposure to the drugs.

Two longitudinal studies have therefore attempted to address this question by assessing the efficacy, safety and tolerability of amodiaquine-artesunate versus AL [55] and amodiaquine plus sulphadoxine-pyrimethamine versus artesunate-amodiaquine versus AL [56] after repeated use.

These studies in Ghanaian and Ugandan children indicated all treatments were efficacious, but the various treatments were associated with slightly different adverse event profiles. For example, treatment with amodiaquine plus sulphadoxine-pyrimethamine had a greater risk compared with AL of anorexia, weakness and subjective fever and a greater risk compared with artesunate-amodiaquine of weakness and subjective fever. Children treated with AL, however, had a higher risk of elevated temperature than those receiving artesunate-amodiaquine [56]. In both studies, repeated administration of AL did not seem to be associated with an increased risk of adverse drug reactions. The study in Ghanaian children also included neurological examinations for children treated with repeated artemisinin-based regimens. This did not identify any abnormal neurological signs that were considered related to artemisinin [55].

## Comparison with other anti-malarial therapies

The safety of the six-dose regimen of AL has been compared with MAS in two Novartis-sponsored, open-label randomized trials [7,8,52], as summarized in Table 1. In these two studies, 314 patients received AL and 105 patients received MAS. Overall, AL was at least as well tolerated as MAS, and had a similar safety profile (Table 6) [52]. Only one patient, who developed urticarial rash, experienced a serious adverse event that was thought to be treatment-related [52]. Independent studies have also compared AL with other anti-malarial therapies as shown in Table 2, Table 3 and Table 4[12-40]. These studies showed that AL was at least as well tolerated as the various comparators (AQSP, ASAQ, ASCD, ASSP, AS plus sulphamethoxypyrazine plus pyrimethamine, atovaquone/proguanil, CQ, CQSP, DP, DNP, MAS, quinine, or SP) used in clinical trials.

**Table 6:** Most frequently reported adverse events that occurred at a frequency of >10% in either group of patients enrolled in comparator studies [7,8,52]

**Adverse event preferred term**	**AL n(%) n = 314**	**MAS n(%) n = 105**
Pyrexia	183 (58.3)	63 (60.0)
Headache	166 (52.9)	46 (43.8)
Dizziness	123 (39.2)	37 (35.2)
Anorexia	108 (34.4)	37 (35.2)
Asthenia	108 (34.4)	33 (31.4)
Arthralgia	99 (31.5)	31 (29.5)
Nausea	82 (26.1)	34 (32.4)
Myalgia	74 (23.6)	19 (18.1)
Sleep disorder	70 (22.3)	30 (28.6)
Chills	67 (21.3)	20 (19.0)
Vomiting	50 (15.9)	22 (21.0)
Abdominal pain	61 (19.4)	20 (19.0)
Palpitation	55 (17.5)	17 (16.2)
Hepatomegaly	48 (15.3)	8 (7.6)
Splenomegaly	40 (12.7)	12 (11.4)
Fatigue	34 (10.8)	8 (7.6)

AL: artemether/lumefantrine; MAS: mefloquine/artesunate

## Safety in pregnancy

Pregnant women with symptomatic malaria, and especially those in their second and third trimesters, are more likely to develop severe malaria than other women [2]. In addition, this is often complicated by pulmonary oedema and hypoglycaemia [2]. Prompt, safe, and effective treatment is, therefore, recommended in this high risk group to reduce maternal mortality, foetal death and premature labour [2]. ACT can be used in uncomplicated malaria in the second and third trimester. The use of ACT in the first trimester should, however, only be considered if they are the only effective treatment available and the benefits outweigh the risks of treatment [2,4]. In severe malaria, ACT is preferred over quinine due to the hypoglycaemia associated with quinine. Until recently there has been limited evidence available to support the use of ACT in early pregnancy; however, McGready *et al *published data on the use of AL in pregnancy in 2009 [57]. These data were from a prospective eight-year study in Thailand on pregnant women exposed to artemisinin antimalarials. In this study, birth outcomes did not differ significantly from community rates of congenital malformations and stillbirth [57]. In addition, data from a prospective observational study to compare the safety of AL and SP in pregnant women treated for symptomatic falciparum malaria was presented at ASTMH Annual Meeting in December 2008 [58]. This study analysed data from 1,001 pregnant women (AL: n = 495; SP: n = 506) and their foetuses/newborns (AL: n = 470; SP: n = 477). There were no clinically relevant differences in perinatal mortality, neonatal mortality, still births, preterm deliveries, gestational age-adjusted low birth weight or birth defects between the two groups.

## Post-marketing experience

As of July 2009, 250 million AL treatments (70% of which were for children) have been delivered to malaria-endemic countries. Post-marketing experience has not identified any new specific safety concerns apart from hypersensitivity and skin reactions (allergies), a recognized class effect for artemisinin derivatives [2].

## Conclusion

This review summarizes some of the safety and tolerability data on AL (Coartem^®^) from published Novartis-sponsored and independently-sponsored clinical trials. Data are available to support the use of a six-dose regimen of AL as a safe and well-tolerated treatment for *P. falciparum *malaria or malaria due to mixed infection including *P. falciparum *in adults, adolescents, children and infants. Indeed, the safety profile of this drug is similar in both adults and children, and many of the reported adverse events are typical of symptoms of malaria or concomitant infections that are commonly seen in these patient populations. Reported adverse events are mainly of mild or moderate severity, with very few serious adverse events reported and even fewer serious adverse events considered related to treatment. In addition, no notable neurological adverse events or cardiac safety concerns were identified in the many studies that examined the safety of AL. As patients (and especially children) often take repeated courses of treatment for malaria, it is reassuring to note that data are available that show repeated administration of AL is not associated with an increased risk of adverse drug reactions, in particular with regard to neurological adverse events. In addition, a thorough review of the clinical data for AL does not identify any potential cardiac safety issues. Data are also available to show that there were no clinically relevant differences in perinatal mortality, neonatal mortality, still birth, pre-term delivery, low birth weight and spontaneous abortion in women exposed to AL compared with SP during pregnancy.

In conclusion, AL is a safe and well-tolerated treatment for *P. falciparum *infections that should help fight malaria. Indeed, as of July 2009, 250 million AL (Coartem^®^) treatments have been delivered and post-marketing experience confirms the favourable safety and tolerability profile for this drug. In addition, this wealth of safety evidence has not identified any safety concerns for AL apart from rare type 1 hypersensitivity reactions, a recognized class effect of artemisinin derivatives [2].

## List of abbreviations

ACT: Artemisinin-based combination therapy; AE: Adverse event; AL: Artemether/lumefantrine; AQ: Amodiaquine; AQSP: Amodiaquine plus sulphadoxine-pyrimethamine; AS: Artesunate: ASAQ: Artesunate plus amodiaquine; ASTMH: American Society of Tropical Medicine and Hygiene; CQ: Chloroquine; CQSP: Chloroquine plus sulphadoxine-pyrimethamine; DNP: Dihydroartemisinin plus napthoquine plus trimethoprim; DP: Dihydroartemisinin-piperaquine; ECG: Electrocardiogram (also known as EKG); hERG: Human ether-a-go-go related gene; IC_50_: Inhibitory concentration 50 (the concentration that gives 50% inhibition); ICH: International Conference on Harmonization; IKr: Rectifying K^+ ^current; MAS: Mefloquine plus artesunate; MedDRA: Medical Dictionary for Regulatory Activities; SAE: Serious adverse event; SP: Sulphadoxine-pyrimethamine; vs.: Versus; WHO: World Health Organization.

## Competing interests

The authors would like to acknowledge that Novartis Pharma AG sponsored this supplement. However, none of the authors works for, or represents in any way, Novartis Pharma AG.

## Authors' contributions

All authors met International Committee of Medical Journal Editors criteria for authorship.
